# Low serum anti-Müllerian hormone is associated with semen quality in infertile men and not influenced by vitamin D supplementation

**DOI:** 10.1186/s12916-023-02782-1

**Published:** 2023-02-28

**Authors:** Rune Holt, Sam Kafai Yahyavi, Ireen Kooij, Christine Hjorth Andreassen, Anna-Maria Andersson, Anders Juul, Niels Jørgensen, Martin Blomberg Jensen

**Affiliations:** 1 Group of Skeletal, Mineral and Gonadal Endocrinology, Department of Growth and Reproduction, Copenhagen University Hospital – Rigshospitalet, Copenhagen, Denmark; 2 Department of Growth and Reproduction, Copenhagen University Hospital – Rigshospitalet, Copenhagen, Denmark; 3 International Center for Research and Research Training in Endocrine Disruption of Male Reproduction and Child Health (EDMaRC), Copenhagen University Hospital – Rigshospitalet, Copenhagen, Denmark; 4 Department of Clinical Medicine, University of Copenhagen, Copenhagen, Denmark; 5 Division of Bone and Mineral Research, HSDM/HMS, Harvard University, Boston, USA

**Keywords:** AMH, Male infertility, Citamin D, Semen quality, Sertoli cell function

## Abstract

**Background:**

Anti-Müllerian hormone (AMH) is released by testicular Sertoli cells and of great importance during fetal male sexual development, but less is known about the role of circulating AMH during adulthood. In vitro studies have shown that vitamin D may induce AMH transcription, but a controlled trial investigating the possible effect of vitamin D on serum AMH has not been conducted in men.

**Methods:**

A single-center, double-blinded, randomized placebo-controlled clinical trial (NCT01304927) conducted in Copenhagen, Denmark. A total of 307 infertile men were included and randomly assigned (1:1) to a single dose of 300,000 IU cholecalciferol followed by 1400 IU cholecalciferol + 500 mg of calcium daily (*n* = 151) or placebo (*n* = 156) for 150 days. Difference in serum AMH was a predefined secondary endpoint. Explorative outcomes were associations between serum AMH and gonadal function in infertile men. The primary endpoint was difference in semen quality and has previously been published.

**Results:**

Infertile men in the lowest AMH tertile had significantly lower sperm concentration (∆_T3-1_ 16 mill/mL (228%); *P* < 0.001), sperm count (∆_T3-1_ 55 million (262%); *P* < 0.001), motile sperm count (∆_T3-1_ 28 million (255%); *P* < 0.001), progressive motile sperm count (∆_T3-1_ 18 million (300%); *P* < 0.001), testis size (∆_T3-1_ 2.7 mL (16%); *P* < 0.001), serum inhibin B (∆_T3-1_ 72 pg/mL (59%); *P* < 0.001), inhibin B/FSH ratio (∆_T3-1_ 48 (145%); *P* < 0.001), and higher FSH (∆_T3-1_ 2.6 (38%); *P* < 0.001) than the tertile of infertile men with highest serum AMH. Vitamin D supplementation had no effect on serum AMH compared with placebo treatment.

**Conclusions:**

In infertile men, low serum AMH is associated with severely impaired gonadal function illustrated by poor semen quality and lower testosterone/LH ratio. Serum AMH in infertile men was not influenced by vitamin D supplementation.

## Background

Anti-Müllerian hormone (AMH) is a glycoprotein belonging to the transforming growth factor β (TGF-β) family. AMH is indispensable for normal sexual differentiation in men and is responsible for regression of the Müllerian duct during the first trimester of male fetal development [1]. AMH is produced by testicular Sertoli cells and reaches the highest levels 3 months postnatally. Serum AMH starts to decrease rapidly at puberty, and adult levels correspond to 3–4% of the concentration found during infancy [2]. Regulation and release of AMH are not fully clarified, although follicle-stimulating hormone (FSH) is a recognized stimulator, while the pubertal increase in testosterone facilitates Sertoli cell maturation and thereby indirectly lowers serum AMH [3]. Serum AMH is positively associated with FSH, inhibin B, and testis size but not with sperm concentration in adult men from the general population [4]. However, only a few studies have investigated the association between serum AMH and semen quality in infertile men and most of these studies are small and have reported conflicting results.

Vitamin D has been suggested to be important for male reproduction and sex steroidogenesis in both animals and humans [5, 6]. A link between vitamin D and AMH has not been thoroughly investigated in men, although a vitamin D response element in the promotor region of *AMH* has been identified, and activated vitamin D induces *AMH* expression in prostate cancer cells [7]. Several studies have investigated the associations between vitamin D status and serum AMH in women but the data show conflicting results as recently reviewed [8]. Here, we studied the possible influence of vitamin D on serum AMH in infertile men by conducting a secondary analysis of a randomized clinical trial testing the effect of high dose vitamin D + calcium versus placebo for 150 days on semen quality in infertile men with vitamin D insufficiency [9]. Now, we show a link between serum AMH and gonadal function in infertile men with impaired semen quality and compare the effect of high-dose vitamin D supplementation versus placebo on serum AMH.

## Methods

### Trial design and participants

The Copenhagen Bone-Gonadal Study is a single-center, double-blinded, randomized clinical trial conducted at the Department of Growth and Reproduction, Copenhagen University Hospital - Rigshospitalet, Copenhagen, Denmark. In brief, infertile men with impaired semen quality and an average duration of infertility of more than 2 years were referred to our andrological center for workup before intracytoplasmic sperm injection (ICSI). Most men had moderate to severe oligospermia, while men with normal sperm count had either very few motile or morphological normal sperm that could explain the long duration of infertility. Inclusion criteria were serum vitamin D level ≤ 50 nmol/L (vitamin D insufficiency) and no serious co-morbidities. Participating men were randomized 1:1 to active or placebo treatment for 150 days. Men in the active group (*n* = 151) initially received an oral bolus of 300,000 IU cholecalciferol, followed by daily supplementation with 1400 IU cholecalciferol and 500 mg calcium (Tablets, Ferrosan/Pfizer, Denmark). The selected intervention was based on an expected increase in serum levels of 25OHD of 50 nmol/L after 150 days of intervention [9]. Men in the placebo group (*n* = 156) received an oral bolus of oil and placebo tablets. Before the andrological examination, all men delivered two semen samples and further two semen samples after 150 days. Differences in serum AMH were a predefined secondary outcome. The primary outcome (semen quality) has previously been published [9].

### Baseline characteristics

The inclusion of study participants has been described in detail previously [9]. Briefly, 307 men out of 1427 referred to our andrological department were included and randomly assigned to receive either vitamin D + calcium or a placebo, and 269 men completed the study. There were no serious adverse events in the group receiving vitamin D supplementation. Baseline characteristics are presented in Table 1.

**Table 1:** Baseline characteristics of included men

Characteristics	(***n***)	Vitamin D + calcium	(***n***)	Placebo	(***n***)	Total cohort
Included men (%)	151	49%	156	51%	307	100
Age (years)	151	34 (30, 39)	156	34 (31, 39)	307	34 (30, 39)
BMI (kg/m^2^)	148	26 (24, 28)	151	26 (24, 28)	299	26 (24, 28)
Current smokers (%)	138	27%	139	22%	277	24%
Cryptorchidism (%)	142	14%	140	16%	282	15%
Testis size (mL)	136	19 (15, 23)	133	18 (15, 21)	269	18 (15, 22)
**Hormones**						
AMH (pmol/L)	147	33 (23, 50)	151	32 (21, 55)	298	33 (22, 54)
Inhibin B (pg/mL)	147	155 (109, 209)	151	145 (99, 185)	298	152 (106, 196)
FSH (U/L)	147	4.3 (2.6, 6.2)	151	4.4 (2.9, 6.1)	298	4.3 (2.7, 6.1)
AMH/FSH ratio	147	8 (4, 17)	151	8 (4, 15)	298	8 (4, 16)
Inhibin B/FSH ratio	147	40 (19, 76)	151	36 (17, 60)	298	37 (18, 67)
LH (U/L)	147	4.2 (3.1, 5.3)	151	3.9 (2.8, 17.2)	298	4.0 (2.9, 5.3)
Testosterone (nmol/L)	147	14 (11, 17)	151	14 (11, 17)	298	14 (11, 17)
Testosterone/LH ratio	147	3.3 (2.5, 4.9)	151	3.6 (2.5, 5.2)	298	3.5 (2.5, 5.0)
Estradiol (pmol/L)	147	96 (82, 113)	151	99 (88, 117)	298	98 (83, 116)
Testosterone/Estradiol ratio	147	149 (123, 185)	151	136 (114, 182)	298	145 (116, 183)
25OHD (nmol/L)	147	43 (29, 59)	153	44 (29, 59)	300	44 (29, 59)
**Semen parameters**						
Abstinence (days)	150	3.8 (3.0, 4.3)	154	3.8 (3.0, 4.5)	304	3.8 (3.0, 4.5)
Semen volume (mL)	150	3.8 (2.7, 5.0)	156	3.6 (2.7, 4.5)	306	3.6 (2.7, 4.7)
Sperm concentration (10^6^/mL)	150	12 (4, 35)	156	14 (3, 44)	306	13 (4, 37)
Sperm count (10^6^)	150	48 (15, 146)	156	48 (10, 132)	306	48 (14, 133)
Motile sperm (%)	144	41 (26, 58)	149	48 (29, 63)	293	45 (27, 61)
Motile sperm (10^6^)	144	23 (5, 69)	149	25 (4, 77)	293	23 (5, 74)
Progressive motile sperm (%)	144	27 (15, 44)	149	34 (17, 49)	293	31 (16, 45)
Progressive motile sperm (10^6^)	144	13 (3, 52)	149	17 (3, 55)	293	17 (3, 54)
Morphological normal sperm (%)	147	2.0 (0.8, 4.0)	150	3.0 (1.5, 6.3)	297	2.5 (1.0, 5.0)
Morphological normal sperm (10^6^)	147	0.9 (0.2, 4.2)	150	1.4 (0.1, 6.7)	297	1.2 (0.2, 5.1)

Data presented as median (25th, 75th percentiles) unless otherwise indicated. Semen parameters are an average of two semen samples delivered before inclusion. Testis size is evaluated by orchidometer and presented as the average of both testes

### Biochemical analyses

Blood samples were collected fasting between 8:00 and 10:00 AM. AMH was measured by a sensitive immunoassay (Immunotech, Beckman Coulter Ltd., Marseilles, France) with an inter-assay coefficient of variation (CV) < 12%. Inhibin B was measured by a specific two-sided enzyme-linked immunoassay (inhibin B genII; Beckman Coulter, Brea, United States of America) with a CV < 11%. FSH and LH were measured by a time-resolved immuno-fluorometric assay (Delfia; Wallac, Turku, Finland) with CV < 4% for both. Testosterone and estradiol levels were measured using radio-immunoassay platforms from Siemens (Los Angeles, USA) and Pantex (Santa Monica, United States of America), with CV < 13% for both. Serum 25-hydroxyvitamin D (25OHD) was measured by isotope dilution liquid chromatography-tandem mass spectrometry with CV < 10%. Except for 25OHD at baseline, all blood analyses were conducted on frozen serum samples from day 1 and day 150 and analyzed after study completion to avoid intraassay variation (year 2015).

### Semen analyses

All semen samples were provided by masturbation on-site in a room close to the laboratory. Two semen samples at baseline were obtained with a mean of 16 days apart. Duration of abstinence, fever, and spillage were self-reported. Semen analyses were conducted as previously described [10]. In short, semen volume was assessed by weight and sperm concentration was determined by the use of a Bürker-Türk hemocytometer. Sperm motility was classified as progressive motile (WHO class A+B), non-progressive motile (class C), or immotile (class D). Sperm morphology was evaluated according to stricter criteria. Almost all men delivered two samples at baseline (95%). Data from men delivering only one sample at baseline are included in the final analysis.

### Statistical analyses

Baseline statistics are presented as median with interquartile range in Tables 1, 2, and 4. Men were stratified according to serum AMH in tertiles and differences were evaluated by the Kruskal-Wallis test for all variables except categorical variables such as currently smoking and cryptorchidism where the chi-square test was used (Table 2). Differences (∆) in serum inhibin B, FSH, testosterone/LH ratio, testosterone/estradiol ratio, testis size, and age between low and high serum AMH tertile are presented as differences in mean and percentage. Subsequently, men were stratified according to serum AMH and serum FSH and differences were evaluated by the Kruskal-Wallis test for all variables, except categorical variables: currently smoking and cryptorchidism where the chi-square test was used (Table 4). Differences (∆) in testosterone/LH ratio and testis size between men with low AMH and high FSH and men with neither low AMH nor high FSH are presented as differences in mean and percentage. All semen parameters presented and used in the statistical analysis are an average of the two semen samples delivered before inclusion. Testis size was evaluated by an orchidometer and is presented as the average of both testes. Multiple linear regression was used to investigate associations between serum AMH, serum inhibin B, serum FSH, and semen parameters after adjustment of relevant confounders: age and BMI. Since we are using an average of two semen samples, we have chosen not to use time of abstinence as a confounder. In the multiple linear regression model sperm concentration, sperm count, motile sperm count, progressive motile sperm count, morphological normal sperm count, and morphological normal sperm (%) were ln-transformed. To make the interpretation of the multiple linear regression analysis more manageable, the β-coefficient and 95% CI were back-transformed [(exp(x)-1) × 100] and presented as percentage (%).

**Fig. 1:**
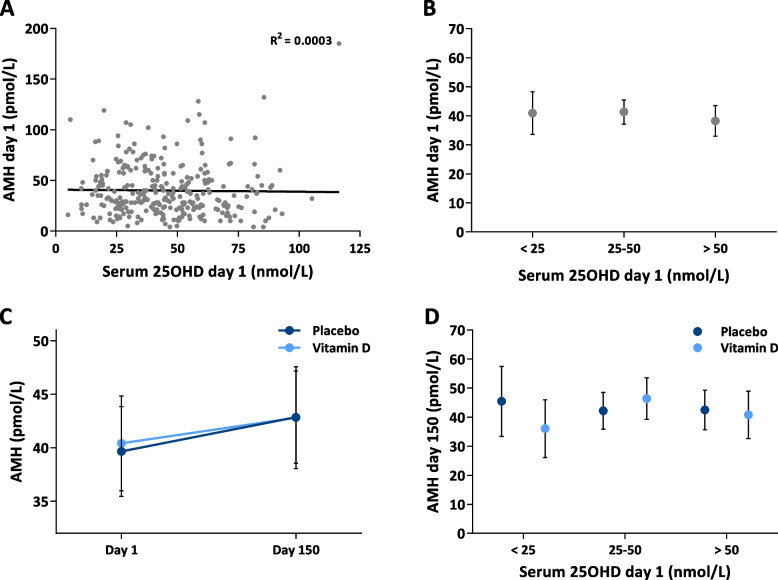
The association between vitamin D and serum AMH at day 1 and after 150 days of supplementation with vitamin D + calcium. **A** Scatter plot of serum 25OHD and AMH at day 1. **B** Serum AMH according to vitamin status at day 1 (< 25 nmol/L: vitamin D deficiency; 25–50 nmol/L: vitamin D insufficiency; > 50 nmol/L: vitamin D sufficiency). **C** Serum AMH at day 1 and after 150 days of intervention in the vitamin D (light blue) and placebo (dark blue) group. **D** Serum AMH after 150 days of intervention stratified according to vitamin status at day 1 (< 25 nmol/L: vitamin D deficiency; 25–50 nmol/L: vitamin D insufficiency; > 50 nmol/L: vitamin D sufficiency). Light blue represents men receiving vitamin D + calcium and dark blue represents men receiving placebo. **B**–**D** are presented as mean ± 95% CI

Association between serum 25OHD and AMH was tested by Pearson’s correlation (Fig. 1A). Differences in serum AMH according to vitamin D status at day 1 (Fig. 1B), before and after intervention (Fig. 1C) and after intervention according to vitamin D status at day 1 (Fig. 1D), were all performed by an unadjusted comparison of means (*t*-test). All statistical analyses were performed by using IBM SPSS Statistics.

## Results

### Serum AMH and testicular function

All men were stratified in tertiles according to serum AMH at day 1 and baseline characteristics are presented in Table 2. Men in the lowest AMH tertile had significantly lower testis size (differences in average between lowest and highest tertile; ∆2.7 mL (16%); *P* < 0.001), serum inhibin B (∆72 pg/mL (59%); *P* < 0.001), inhibin B/FSH ratio (∆48 (145%); *P* < 0.001), testosterone/LH ratio (∆0.7 (20%); *P* = 0.007), testosterone/estradiol ratio (∆22 (16%); *P* = 0.007) and higher serum FSH (∆2.6 U/L (38%); *P* < 0.001) compared with men in the highest AMH tertile. Moreover, men in the lowest AMH tertile were significantly older than men in the highest tertile (∆3.9 years (11%); *P* < 0.001). There were no differences in serum testosterone, estradiol, LH, 25OHD, BMI, or cryptorchidism (%) between the groups. The differences in testis size and reproductive hormones were supported by showing lower sperm production (differences in the median between lowest and highest tertile; sperm concentration (∆16 million/mL (228%); *P* < 0.001), sperm count (∆55 million (262%); *P* < 0.001), motile sperm count (∆28 million (255%); *P* < 0.001), progressive motile sperm count (∆18 million (300%); *P* < 0.001) and morphological normal sperm count (∆1.3 million (260%); *P* = 0.006) in the low versus high AMH tertile group. There was no difference between sperm motility (%), progressive motility (%), or morphology (%) (Table 2).

**Table 2:** Characteristics of included men stratified in tertiles according to serum A﻿MH﻿ at day 1

	AMH lowest tertile (*n*=100)	AMH median tertile (*n*=99)	AMH highest tertile (*n*=99)	*P-value
Age (years)	37 (32, 40)	34 (30, 38)	32 (29, 37)	**<0.001**
BMI (kg/m^2^)	26 (24, 29)	26 (23, 28)	26 (23, 28)	0.154
Smoking (%)	27 (%)	23 (%)	24 (%)	0.734
Cryptorchidism (%)	18%	15%	13%	0.715
Testis size (mL)	17 (14, 20)	19 (16, 22)	20 (16, 25)	**<0.001**
AMH (pmol/L)	17 (13, 22)	33 (28, 39)	64 (54, 78)	-
Inhibin B (pg/mL)	116 (82, 148)	158 (117, 199)	178 (140, 236)	**<0.001**
FSH (U/L)	5.2 (3.7, 8.0)	4.1 (2.9, 5.4)	3.3 (2.2, 5.4)	**<0.001**
AMH/FSH ratio	3.4 (2.0, 4.7)	8.5 (5.6, 12.6)	19.5 (11.1, 31.0)	-
Inhibin B/FSH ratio	21 (10, 42)	39 (23, 70)	51 (31, 93)	**<0.001**
LH (U/L)	4.3 (3.1, 6.0)	3.9 (3.0, 5.0)	3.8 (2.8, 5.3)	0.149
Testosterone (nmol/L)	13 (11, 16)	14 (11, 18)	15 (12, 18)	0.064
Testosterone/LH ratio	3.0 (2.2, 4.5)	3.6 (2.6, 5.4)	3.7 (2.8, 5.1)	**0.007**
Estradiol (pmol/L)	100 (88, 117)	97 (86, 112)	97 (77, 117)	0.101
Testosterone/estradiol ratio	130 (99, 170)	148 (115, 184)	154 (127, 187)	**0.007**
25OHD (nmol/L)	50 (30, 61)	42 (29, 55)	40 (28, 59)	0.096
Abstinence (days)	3.6 (3.0, 4.3)	3.8 (3.0, 4.6)	3.8 (3.0, 4.6)	0.516
Semen volume (mL)	3.5 (2.6, 4.5)	3.8 (2.6, 4.9)	3.7 (3.0, 4.8)	0.321
Sperm concentration (10^6^/mL)	7 (2, 23)	14 (5, 40)	23 (7, 53)	**<0.001**
Sperm count (10^6^)	21 (7, 81)	50 (20, 145)	76 (25, 192)	**<0.001**
Motile sperm (%)	39 (23, 53)	47 (29, 60)	44 (28, 63)	0.096
Motile sperm (10^6^)	11 (2, 38)	27 (7, 76)	39 (8, 102)	**<0.001**
Progressive motile sperm (%)	27 (23, 41)	31 (17, 44)	31 (16, 52)	0.114
Progressive motile sperm (10^6^)	6 (1, 25)	20 (3, 57)	24 (4, 80)	**<0.001**
Morphological normal sperm (%)	2.3 (1.0, 5.5)	2.5 (1.0, 4.8)	2.5 (1.0, 5.8)	0.808
Morphological normal sperm (10^6^)	0.5 (0.1, 2.6)	1.5 (0.2, 4.5)	1.8 (0.3, 8.1)	**0.006**

Data presented as median (25th, 75th percentiles) unless otherwise is indicated. *P-value: Kruskal-Wallis test for all, except for smoking and cryptorchidism where the chi-squared test was used. Semen parameters are presented as an average of two semen samples delivered before inclusion. Testis size is evaluated by orchidometer and presented as the average of both testes

Men with low serum AMH had impaired gonadal function compared with men having higher serum AMH. In accordance, serum AMH was positively associated with sperm concentration (*β*: 1.5%; *P* < 0.001), sperm count (*β*: 1.6%; *P* < 0.001), motile sperm count (*β*: 1.7%; *P* < 0.001), progressive motile sperm count (*β*: 1.8%; *P* < 0.001) and morphological normal sperm count (*β*: .2.2%; *P* < 0.001) (Table 3). There was no association between serum AMH and sperm motility (%) or progressive motility (%) although morphology (%) was borderline significant (*β*: 0.4%; *P* = 0.058). Overall, serum FSH was negatively associated with most semen quality variables. Serum FSH were negatively associated with morphology (%) (*P* = 0.049), while a similar link with sperm motility (%), and progressive motility (%) was not statistically significant (*P*=0.076 and *P*=0.085, respectively). In comparison, serum AMH and inhibin B concentrations were not associated with morphology (%), sperm motility (%) or progressive motility (%) (Table 3).

**Table 3:** Association between markers of Sertoli cell function (serum AMH, ﻿inhibin B, and FSH) and semen quality

	Serum AMH (pmol/L)			Serum inhibin B (pg/mL)			Serum FSH (U/L)		
	*β*	95% CI	*P*	*β*	95% CI	*P*	*β*	95% CI	*P*
Sperm concentration (10^6^/mL)^a^	1.5%	0.7–2.4%	**< 0.001**	1.0%	0.8–1.3%	**< 0.001**	−16.2%	−20.3 to −12.0%	**< 0.001**
Sperm count (10^6^)^a^	1.6%	0.8–2.5%	**< 0.001**	1.0%	0.8–1.3%	**< 0.001**	−16.0%	−20.2 to −11.6%	**< 0.001**
Motile sperm (%)	0.1%	−0.0–0.2%	0.276	0.0%	−0.0–0.1%	0.294	−0.6%	−1.2–0.1%	0.076
Motile sperm (10^6^)^a^	1.7%	0.8–2.7%	**< 0.001**	0.9%	0.6–1.2%	**< 0.001**	−15.6%	−20.3 to −10.7%	**< 0.001**
Progressive motile sperm (%)	0.1%	−0.0–0.2%	0.152	0.0%	−0.0–0.0%	0.243	−0.5%	−1.1–0.1%	0.085
Progressive motile sperm (10^6^)^a^	1.8%	0.8–2.7%	**< 0.001**	1.0%	0.6–1.3%	**< 0.001**	−16.1%	−21.0 to −11.0%	**< 0.001**
Morphological normal sperm (%)^a^	0.4%	−0.0–0.9%	0.058	0.1%	−0.1–0.2%	0.301	−3.0%	−5.9–0.0 %	**0.049**
Morphological normal sperm (10^6^)^a^	2.2%	1.2–3.3%	**< 0.001**	1.0%	0.7–1.3%	**< 0.001**	−17.6%	−22.6 to −12.4%	**< 0.001**

All analyses are adjusted for age and BMI. β, regression coefficient. ^a^Data log-transformed, for additional details see Statistical analyses

Since AMH is a product and FSH a stimulator of Sertoli cells, we evaluated whether a combination of serum AMH and FSH would be superior to either hormone alone. Men with AMH in the lowest tertile and FSH in the highest tertile corresponds to 16% of the cohort, who had a significantly lower sperm production (differences in median; sperm concentration (∆19 million/mL (475%), sperm count (∆70 million (500%), motile sperm count (∆33 million (550%), progressive motile sperm count (∆20 million (500%) and morphologically normal sperm count (∆1.9 million (950%); *P* < 0.001 for all) compared to men with neither AMH in the lowest or FSH in the highest tertile. Moreover, men with AMH in the lowest tertile and FSH in the highest tertile had a significantly lower sperm motility (%) and tended to have a lower progressive motility (%) (∆6%, and ∆4%; difference in media, *P* = 0.042 and 0.067, respectively) compared to men with neither AMH in the lowest or FSH in the highest tertile. There were no differences in morphology (%) between the groups (Table 4). Noteworthy, testis size (differences in average; ∆4.5 mL (29%); *P* < 0.001) and testosterone/LH ratio (∆1.5 (50%); *P* < 0.001) were significantly lower in men with low AMH and high FSH compared with men having neither low AMH nor high FSH.

**Table 4:** Characteristics of included men stratified according to serum AMH and FSH at day 1

	Low AMH and high FSH (*n*=49)	Low AMH or high FSH (*n*=102)	Neither low AMH nor high FSH (*n*=147)	*P-value
Age (years)	38 (34, 42)	34 (30, 39)	33 (29, 38)	**<0.001**
BMI (kg/m^2^)	26 (23, 29)	25 (24, 29)	26 (23, 28)	0.891
Smoking (%)	21 (%)	30 (%)	22 (%)	0.278
Cryptorchidism (%)	23%	18%	11%	0.070
Testis size (mL)	15 (13, 19)	18 (15, 21)	20 (17, 25)	**<0.001**
Testosterone (nmol/L)	14 (11, 16)	14 (11, 17)	14 (11, 17)	0.603
Testosterone/LH ratio	2.4 (1.6, 3.9)	3.3 (2.4, 4.4)	4.0 (2.8, 5.8)	**<0.001**
Estradiol (pmol/L)	98 (87, 115)	102 (82, 121)	97 (83, 113)	0.441
Testosterone/estradiol ratio	130 (113, 184)	143 (113, 180)	149 (120, 186)	0.265
Abstinence (days)	3.8 (3.3, 4.0)	3.5 (3.0, 4.3)	3.8 (3.0, 4.8)	0.381
Semen volume (mL)	4.0 (2.6, 5.0)	3.4 (2.6, 4.4)	3.8 (2.9, 5.0)	0.066
Sperm concentration (10^6^/mL)	4 (1, 9)	10 (2, 26)	23 (8, 55)	**<0.001**
Sperm count (10^6^)	14 (3, 36)	39 (8, 85)	84 (28, 189)	**<0.001**
Motile sperm (%)	32 (23, 53)	46 (27, 57)	47 (29, 64)	**0.042**
Motile sperm (10^6^)	6 (1, 17)	17 (3, 41)	39 (10, 103)	**<0.001**
Progressive motile sperm (%)	24 (13, 40)	32 (13, 44)	31 (17, 51)	0.067
Progressive motile sperm (10^6^)	4 (1, 14)	10 (2, 30)	24 (4, 80)	**<0.001**
Morphological normal sperm (%)	2.0 (0.8, 3.8)	2.1 (1.0, 4.9)	2.8 (1.4, 6.0)	0.182
Morphological normal sperm (10^6^)	0.2 (0.1, 1.0)	0.6 (0.1, 2.9)	2.1 (0.4, 8.4)	**<0.001**

Data presented as median (25th, 75th percentiles) unless otherwise indicated. *P-value: Kruskal-Wallis test for all, except for smoking and cryptorchidism where the chi-squared test was used. Semen parameters are presented as an average of two semen samples delivered before inclusion. Testis size is evaluated by orchidometer and presented as the average of both testes

### Vitamin D supplementation and serum AMH

At baseline, all included men were vitamin D insufficient with a mean serum 25OHD of 35 nmol/L. On day 1, the mean serum 25OHD had increased to 45 nmol/L despite no treatment received. After 150 days of intervention, men randomized to vitamin D and calcium had a significant average 93% increase in serum 25OHD from mean 46 nmol/L to 89 nmol/L, while men randomized to placebo had a non-significant average 13% increase in serum 25OHD from mean 45 nmol/L to 51 nmol/L. On day 1, the vitamin D and the placebo group had an average serum AMH of 40 pmol/L and there were no significant associations between serum 25OHD and serum AMH (Fig. 1A). Furthermore, serum AMH was not different when stratified according to vitamin D status at day 1, vitamin D deficiency (< 25 nmol/L; AMH = 41 pmol/L), insufficiency (25–50 nmol/L; AMH = 41 pmol/L) or sufficiency (> 50 nmol/L; AMH = 38 pmol/L) (Fig. 1B). Vitamin D and placebo-treated men experienced both a small non-significant increase in serum AMH from mean 40 pmol/L to 43 pmol/L (Fig. 1C). Vitamin D supplementation induced no change or difference in serum AMH compared with placebo when stratified in subgroups according to vitamin D status at day 1 (Fig. 1D). The effect of vitamin D supplementation on serum FSH and serum inhibin B have been published in the primary manuscript [9].

## Discussion

This study shows that low serum AMH is a marker for poor gonadal function illustrated by impaired semen quality and lower serum inhibin B, inhibin B/FSH ratio, testosterone/LH ratio, and higher serum FSH in a well-characterized cohort of infertile men. AMH is produced by Sertoli cells, but its biological function in postnatal life is largely unknown. This study suggests that low serum AMH may reflect a poor Sertoli cell function, which is supported by the lower serum inhibin B, higher FSH, and lower inhibin/FSH ratio in men having the lowest serum AMH with a threshold at 26 pmol/L. The observed low inhibin B/FSH ratio and impaired semen quality in men with the lowest serum AMH suggest that low AMH reflects inappropriate Sertoli cell function, which may be unable to support an adequate number of germ cells. This suggestion is supported by the concomitant threefold lower sperm quantity and smaller testis size in men with low serum AMH. Testis size is largely determined by the number of germ cells, which indicates that low serum AMH may be a marker of the function of seminiferous tubules and Sertoli-germ cell interaction. Still, low AMH may also reflect Leydig cell function although serum testosterone, estradiol, and LH were not different between men stratified according to AMH tertiles. Instead, the testosterone/LH ratio was lower, which suggests that men with low AMH in addition to impaired Sertoli cell function may have impaired Leydig cell function.

Ideally, the observed differences in reproductive hormones between AMH groups could be supported by evaluation of testis histology, but despite this limitation, it is unlikely that men with low serum AMH have more immature Sertoli cells as they in theory would produce higher levels of AMH than mature Sertoli cells [3, 11]. Moreover, our findings are in accordance with a recent study showing that serum AMH is positively associated with sperm production, serum FSH, and serum inhibin B in a cohort of normozoospermic men [12]. Several other studies have measured serum AMH in both fertile and infertile men but most of these cohorts are relatively small. Our data are at least in part in accordance with a large cross-sectional study of 970 young men from the general population with unknown fertility potential showed that men with low serum AMH (< 30 pmol/L) had significantly lower testis size, serum inhibin B, inhibin B/FSH ratio and testosterone/estradiol ratio than men with higher serum AMH [4]. Interestingly, these young men from the general population had higher serum AMH (using same assay) and better semen quality than our cohort of infertile men. Moreover, only infertile men with the lowest AMH had severely impaired gonadal function, which indicates that low AMH in infertile men may be considered a risk factor for severely impaired gonadal function, but this may not be the case for fertile men. We can only speculate why low serum AMH may be linked with more detrimental gonadal function, particularly in infertile men, however, infertile men may have less compensatory capacity to accommodate for lower AMH than fertile men who as a group have lower serum FSH levels and thus require less stimulation of the Sertoli cells. The large study of young men from the general population found no link between serum AMH and sperm production, which is in accordance with other studies in fertile men [4, 13, 14]. Still, Tüttelmann et al found that serum AMH was positively associated with sperm concentration in men with the lowest sperm concentration (0.1-7.9 million/mL) [13]. Moreover, in a mixed group of fertile and infertile men, Appasamy et al found serum AMH was positively associated with sperm concentration, but the positive link was not corroborated by others [15, 16]. Serum AMH has been reported to be lower in infertile men compared with fertile men [17–19] but other studies have not been able to support this [13, 15, 16, 20], which highlights that serum AMH preferentially may be used prognostically in infertile men rather than as a diagnostic marker of infertility. One explanation for the discrepancy could be the heterogenous caused for male infertility and some infertile men may have higher serum AMH levels due to more immature Sertoli cells that would limit the clinical applicability of a “high” or “normal” serum AMH. The testicular origin of AMH in seminal plasma is supported by the undetectable levels in men with obstructive azoospermia [21, 22]. Prepubertal AMH is only secreted into circulation, but after puberty AMH is released preferentially towards the lumen of the seminiferous tubules, resulting in higher concentrations in the seminal plasma than in serum [3]. The associations between AMH levels in seminal plasma and semen parameters have been investigated previously and seminal plasma AMH is positively associated with total sperm count and sperm concentration [14, 22, 23], which supports that high AMH is linked with better gonadal performance. Furthermore, fertile men have significantly higher seminal plasma AMH compared with men with impaired semen quality [21–24].

Serum inhibin B and FSH were also associated with sperm production, and serum FSH was stronger associated with sperm production compared with serum AMH and serum inhibin B. FSH is known to be a potent regulator of sperm production in both mice and humans. However, the association between serum AMH and sperm production was not inferior to the link found with serum inhibin B and sperm production. Furthermore, serum AMH was strongly associated with serum inhibin B and serum FSH, which are in line with previous studies [4, 12, 14] and support that serum AMH is a marker of Sertoli cell function. We found that combined low levels of serum AMH and high levels of serum FSH tend to be a better marker for semen quality compared to AMH alone, though only found in up to 16% of infertile men referred for andrological workup prior to ICSI. A combined low serum AMH and high serum FSH as a worse prognostic sign in infertile men, may not be a surprise, but it could be of clinical relevance to measure both hormones under the initial andrological workup.

In vitro data indicate a regulatory role of vitamin D for AMH production [7], but our study is unable to support this notion as the observed correction of vitamin D insufficiency did not affect serum AMH. Moreover, there was no change in serum AMH over time in the vitamin D-treated men despite a marked increase in serum 25OHD and 1,25(OH)_2_D_3_ after the intervention that clearly differed from the placebo-treated men [9]. Several studies have investigated and reported positive associations between serum AMH and vitamin D status, although most of these studies have been conducted on women. A meta-analysis from 2020 concluded that vitamin D supplementation increased serum AMH levels in non-PCOS women, while it decreased serum AMH levels in PCOS women [8]. A recent study conducted in vitamin D-deficient women with diminished ovarian reserve showed that vitamin D supplementation significantly increased serum AMH [25]. Only a few studies have investigated the relationship between vitamin D and serum AMH in men. One study showed a positive association between vitamin D status and serum AMH in men between 54 and 93 years of age but not in prepubertal boys [26]. Another study in men with chronic kidney disease showed a positive association between vitamin D status and serum AMH [27]. In our cohort of infertile men, there was no association between vitamin D status and serum AMH nor any effect of vitamin D supplementation on serum AMH. Vitamin D supplementation had no effect on serum AMH even in men with vitamin D deficiency from the start, which suggests that vitamin D supplementation has none or negligible importance for serum AMH in infertile men.

We propose that serum AMH in infertile men is a marker for Sertoli cell health and function that ultimately, may be used to select the infertile men who will benefit from a Sertoli cell stimulator such as FSH, clomiphene or as recently suggested a receptor activator of nuclear factor kappa-β ligand (RANKL) inhibitor [28]. Mechanistic studies have shown that genetic inhibition or use of a RANKL inhibitor stimulated AMH production in both mice and infertile men [29, 30]. More importantly, infertile men with low serum AMH had a lower increase in sperm production following treatment with RANKL inhibitor compared with infertile men having higher serum AMH levels that had a huge increase in sperm production, indicating a potential use of serum AMH to distinguish poor from good responders and potentially be used to select suitable candidates for the stimulatory treatment of male infertility [28, 29]. Clearly, these studies have to be extended and repeated but future studies are needed to determine whether serum AMH alone or in combination with other gonadal markers such as inhibin B, testosterone, FSH, or insulin-like peptide 3 (INSL3) can be used to select infertile men suitable for medical interventions to improve sperm production and male fertility.

## Conclusions

This study shows that low serum AMH may serve as a marker for impaired Sertoli cell function and poor sperm production in infertile men. Serum AMH was not associated with vitamin D status in infertile men and serum AMH cannot be modified by vitamin D supplementation, which questions the impact of vitamin D on serum AMH in infertile men.

## Data Availability

The datasets used and/or analyzed during the current study are available from the corresponding author on reasonable request.

## Abbreviations

25OHD: 25-hydroxyvitamin D
AMH: Anti-Müllerian hormone
BMI: Body mass index
FSH: Follicle stimulating hormone
LH: Luteinizing hormone
RANKL: Receptor activator of nuclear factor kappa-β ligan
TGF-β: Transforming growth factor β
VDR: Vitamin D receptor
